# The effectiveness of a group-based computerized HIV/STI prevention intervention for black women who use drugs in the criminal justice system: study protocol for E-WORTH (Empowering African-American Women on the Road to Health), a Hybrid Type 1 randomized controlled trial

**DOI:** 10.1186/s13063-018-2792-3

**Published:** 2018-09-10

**Authors:** Karen Johnson, Louisa Gilbert, Timothy Hunt, Elwin Wu, Lisa Metsch, Dawn Goddard-Eckrich, Stanley Richards, Rick Tibbetts, Jessica C. Rowe, Milton L. Wainberg, Nabila El-Bassel

**Affiliations:** 1 University of Alabama School of Social Work, Little Hall, 670 Bonner Drive, Tuscaloosa, AL 35401 USA; 2 Department of Sociomedical Sciences, Mailman School of Public Health, Columbia University, 722 West 168th Street, New York, NY 10032 USA; 3 Social Intervention Group, Columbia University Teacher’s College, 1255 Amsterdam Avenue, New York, NY 10027 USA; 4 The Fortune Society, 625 West 140th Street, New York, NY 10031 USA; 5 New York City Department of Probation, 210 Joralemon Street, Brooklyn, NY 11201 USA; 6 Center for Teaching and Learning, Columbia University, Lewisohn Hall, 2970 Broadway #603, New York, NY 10027 USA; 7 Columbia University / New York State Psychiatric Institute, 1051 Riverside Drive, #24, New York, NY 10032 USA; 8 Columbia University School of Social Work, Social Intervention Group, 1255 Amsterdam Avenue, New York, NY 10027 USA

## Abstract

**Background:**

This paper describes the study protocol of a hybrid type I randomized controlled trial that evaluates the effectiveness and cost-effectiveness of implementing Empowering African-American Women on the Road to Health (E-WORTH), an Afrocentric, group-based, computerized human immunodeficiency virus (HIV)/sexually transmitted infection (STI) prevention intervention for controlled substance-using black women in community corrections settings in New York City.

**Methods/design:**

We provide an overview of E-WORTH’s hybrid type I design, which is guided by the Consolidated Framework for Implementation Research (CFIR). E-WORTH combines HIV/STI and intimate partner violence (IPV) prevention components and tests the comparative effectiveness of E-WORTH and streamlined HIV testing versus streamlined HIV testing alone in decreasing biologically confirmed HIV and STI incidence, sexual risk, and IPV, as well as in improving access to HIV and STI prevention services and care.

**Discussion:**

This paper provides an overview of E-WORTH’s intervention protocol and serves as a framework for using hybrid type I designs, guided by the CFIR conceptual framework, to evaluate HIV/STI and IPV prevention interventions in community corrections settings. We discuss how E-WORTH’s hybrid type I design advances implementation science through its effectiveness and cost-effectiveness aims as well as through a mixed-methods study that evaluates multilevel theory-driven factors (structural, organizational, staffing, and client) guided by the CFIR that influences the implementation of E-WORTH in a criminal justice setting. This study also addresses the novel challenges and opportunities of implementing an intervention that targets specific racial subgroup(s) in a community corrections setting that services all populations, implementing a group-based intervention with technological components in such settings, and employing community-based participatory research strategies to guide recruitment and retention efforts.

**Trial registration:**

ClinicalTrials.gov, NCT02391233. Registered on 17 March 2015.

**Electronic supplementary material:**

The online version of this article (10.1186/s13063-018-2792-3) contains supplementary material, which is available to authorized users.

## Background

Human immunodeficiency virus (HIV) and other sexually transmitted infections (STIs) disproportionately affect criminal justice-involved black women in the United States who use drugs [1–6]. Despite more than 30 years of targeted prevention research and public health interventions, rates of HIV infection and STIs among black women remain disturbingly high when compared with women of other races and ethnicities [7–9]. Black women account for 60% of all new HIV infections among women, although they represent approximately 13% of the general female population [7, 8]. Specifically, when compared with white women, black women have 5.4 times the rate of chlamydia, 8.8 times the rates of primary and secondary syphilis, and 9.7 times the rate of gonorrhea [7].

Black women are also highly represented in multiple sectors of the criminal justice system [10–13]. They are more than twice as likely to become imprisoned as white women, and drug-related crimes remain a leading cause of arrest and incarceration [10–12, 14]. However, the vast majority of criminal justice-involved women are not currently incarcerated. Of the 1.2 million women convicted of a crime, only 15% are incarcerated; the remaining 85%, or approximately 1 million women each year, are sentenced to some form of community corrections (probation, parole, drug treatment court, alternative to incarceration) [13, 15, 16]. When compared with justice-involved women of other ethnicities and racial backgrounds, black women serving sentences in the community are more likely to engage in a variety of risky sexual behaviors, including having unprotected sex with paying partners, using illicit substances before and during sexual encounters, and trading sex for drugs [17–21].

In light of the aforementioned considerations, the importance of providing testing and HIV/STI prevention services to justice-involved populations in community corrections settings has gained attention in the past decade [2, 4, 15, 16, 22–24]. Increasing the number and quality of health services in these settings has been positively linked to a greater use of health care and a reduction in future justice system involvement [24–27]. Furthermore, although few in number, HIV/STI prevention interventions delivered in community justice settings have been found to increase treatment adherence, HIV and STI testing, and protected sex acts and to decrease risky sexual behaviors [3, 28–33]. A randomized controlled trial conducted with 1263 probationers and parolees found that participants who were assigned the option to receive on-site rapid HIV testing at probation offices were significantly more likely to get tested than participants assigned to an off-site community HIV testing site, owing to barriers associated with travel, court mandates, and stigma [31]. A 2009 randomized trial examining the efficacy of brief negotiation interviewing when compared with usual educational activities (*N* = 212) found that participants randomized to the brief negotiation interviewing treatment arm had significantly higher rates of HIV testing than those assigned to the control condition [28]. In addition, participants in the intervention arm were more likely to contemplate changing risky behavior. In a multisite randomized control trial, Nydegger and colleagues administered a 1-hour HIV educational group session to increase intention to use condoms among nonviolent drug offenders participating in mandatory drug diversion programs in Southern California (*N* = 143) [32]. Participants assigned to the experimental arm reported stronger implementation intentions to use condoms than those in the control arm (*p* < 0.001).

Meyer and colleagues conducted an exhaustive review of the literature published in the United States to identify optimal strategies for delivering evidence-based HIV interventions in criminal justice settings or to justice-involved populations [24]. Importantly, the researchers found that a mere 16% of all interventions identified (7 of a total of 42) were delivered in community corrections settings. Only one of the seven, the one from which the intervention in the present study was adapted (Women on the Road to Health [WORTH]), was explicitly designed for women [24]. Substantial research has established the effectiveness of HIV/STI prevention interventions that are specifically tailored to black persons, women, and black women through the use of culturally congruent and gender-congruent themes and relevant peer group facilitators [34–36]. To date, however, despite continuing disproportionately high rates of STIs, rates of criminal justice involvement, and illicit substance use [18, 20, 37], there are no prevention interventions tailored to the needs of drug-using black women involved in the criminal justice system and none that have been tested in hybrid type I effectiveness trials.

Intertwined with the HIV/STI epidemic among substance-using black women who are involved in the criminal justice system is the co-occurring epidemic of intimate partner violence (IPV) [38–40]. Approximately 70% of substance-using black women in the 2009 trial of the multimedia WORTH intervention reported experiencing physical, sexual, and/or injurious IPV in their lifetime [21]. Abundant research has found that experiencing IPV is strongly associated with engaging in unprotected sex, having multiple sexual partners, testing positive for HIV/STIs, and failure to access and adhere to HIV treatment [41–45]. The multimedia WORTH intervention was found to be efficacious in reducing physical, sexual, and injurious IPV at the 12-month follow-up compared with the Wellness Promotion control group [21].

E-WORTH (Empowering African-American Women on the Road to Health) fills these gaps. (Although the term African-African is used in the title of the intervention, E-WORTH is designed for all women who self-identify as black.) E-WORTH extends the reach of HIV/STI prevention efforts and the original scope of WORTH by targeting substance-using black women via the venue of community corrections. Adapted from the WORTH intervention, E-WORTH is imbued with the Afrocentric themes of risk and resiliency and is designed to reach the large and highly vulnerable number of justice-involved black women who remain at very high risk for HIV/STIs and IPV. E-WORTH, and the 2009 Multimedia WORTH intervention, includes a computerized self-paced IPV screening, brief intervention, and referral to treatment tool using the Screening, Brief Intervention, and Referral to Treatment tool (SBIRT) [3, 21].

This paper describes a randomized controlled hybrid effectiveness-implementation type I trial that blends effectiveness and implementation science, as well as the trial methodology used to evaluate the effectiveness and cost-effectiveness of delivering E-WORTH [46]. The aim of the study is to evaluate the effectiveness of delivering E-WORTH in a real-world setting and combining E-WORTH and streamlined HIV testing versus streamlined HIV testing alone as a comparison condition to reduce HIV/STIs among substance-using black women in community corrections settings. The streamlined HIV testing component used in both intervention arms is a very brief HIV information intervention provided with rapid HIV testing. Streamlined testing has been found to be as effective in increasing HIV testing rates and lowering behavioral risks with at-risk populations as more intensive HIV testing and counseling [47, 48].

We provide an overview of the study protocol and discuss the innovative approaches used in evaluating the effectiveness and cost-effectiveness aims. We describe the challenges and opportunities of using of technology to implement E-WORTH in community corrections settings. We also provide an overview of our use of community-based participatory research (CBPR) strategies to solicit feedback regarding the adaptation and implementation of the study from key community partners, including former study participants who meet study criteria; criminal justice advocates; service providers from harm reduction, substance abuse treatment, and criminal justice organizations; and government representatives from community corrections and the New York City Department of Health. E-WORTH employs mixed methods to identify the multilevel structural, community, organizational, staffing, and client factors that influence the delivery of E-WORTH in community corrections settings. In addition, we highlight the use of Social Cognitive Theory [49, 50] and Empowerment Theory [51] to guide the intervention. We also review the use of the Consolidated Framework for Implementation Research (CFIR) [52] conceptual framework to guide the adoption, implementation, and fidelity of the intervention.

WORTH is a Centers for Disease Control and Prevention best practice intervention that was developed for incarcerated substance-using women [53]. The original WORTH intervention has been adapted multiple times and found to be efficacious in reducing condomless sex and physical and sexual IPV among women who use drugs in community corrections settings, including but not limited to those supervised by New York City Department of Probation as well as women in drug treatment settings [3, 5, 21, 54]. In the 2009 trial of the WORTH intervention (*N* = 337), 17% of the 221 black female participants tested positive for HIV, and 30% tested positive for at least one STI [21]. Rates of HIV infection among white and Latina women in the original WORTH trial were significantly lower [3]. The alarmingly high HIV rate found among black participants in the original WORTH study is comparable to rates in Sub-Saharan Africa and is suggestive of a concentrated epidemic among black women who use drugs and are also engaged in justice system supervision. The New York City Department of Probation played a key role in providing access to participants for the 2009 trial in various probation sites. The New York City Department of Probation also provided support in grounding 2009 WORTH study findings in terms of global HIV and STI infection rates among community corrections populations as compared with the general population. In addition, because E-WORTH is designed to be delivered in low-resource probation settings that are overburdened with high caseloads and lack professional staffing and sufficient resources to implement HIV/STI and IPV prevention services, we highlight the ways in which E-WORTH advances the continuum of cost-effective HIV prevention, testing, and treatment interventions that may be scaled up in community corrections settings nationwide.

### Overview of study design and aims

This hybrid effectiveness trial aims to enroll 420 black women who use controlled substances and must undergo supervision at community corrections sites in New York City. Eligible women are randomly assigned to (1) E-WORTH, consisting of an individual streamlined HIV testing session followed by a four-session group-based multimedia HIV/STI prevention intervention (E-WORTH); or (2) an individual streamlined HIV testing session alone (streamlined HIV testing), which will serve as the comparison condition. Both conditions are delivered by community corrections providers. Repeated assessments occur at baseline and 3, 6, and 12 months postintervention. We conduct repeated assessments and qualitative interviews on multilevel factors that may influence the effectiveness of both interventions on study outcomes, and these are conducted with probation providers delivering the interventions, frontline probation staff, and probation administrators. This study has the following specific aims:

1. To test the comparative effectiveness of E-WORTH plus streamlined HIV testing versus streamlined HIV testing alone on primary outcomes of decreasing biologically confirmed HIV and other STIs and the number and proportion of unprotected sex acts at the 12-month follow-up and secondary outcomes of reducing drug use, increasing use of drug treatment, linkage to HIV care and antiretroviral therapy adherence (for HIV-positive participants), and decreasing incidence of IPV and recidivism.
2. To test if the effectiveness of E-WORTH on study outcomes is moderated by client characteristics (e.g., client subgroups defined by sociodemographic and psychosocial characteristics)
3. To estimate the costs and comparative cost-effectiveness of E-WORTH plus streamlined HIV testing versus streamlined HIV testing alone on STI infection rates, drug use, and projected number of HIV cases averted at the 12-month follow-up.
4. To examine qualitatively and quantitatively how multilevel theory-driven factors may influence the fidelity of implementation and effectiveness of E-WORTH and streamlined HIV testing on study outcomes.

### Theoretical and conceptual framework

E-WORTH’s intervention is informed by a theoretical framework that incorporates Empowerment Theory [51] and Social Cognitive Theory [50, 55]. The study’s implementation science conceptual framework is guided by the CFIR.

#### Theory

E-WORTH aims to elicit intrinsic motivation for reducing HIV/STI risks, increase positive self-talk, enhance sexual negotiation skills to refuse unsafe sex, develop strategies and set goals for reducing sexual and drug risk behaviors, enhance social support networks, improve relationship safety and reduce risks for experiencing IPV, and identify and link to services (i.e., general health and mental health referrals, HIV care, and drug treatment programs). Consistent with the tenets of Empowerment Theory [51] and Social Cognitive Theory [50, 55], E-WORTH provides participants with opportunities to develop self-efficacy and practice mastery of sexual negotiation and problem-solving skills to reduce risky behaviors through interactive group activities. In light of the disproportionately high percentage of black women who are involved with the justice system, E-WORTH also infuses empowerment principles by raising awareness of structural forces of sexism, racism, and institutionalized oppression that originate from slavery and historical responses of resilience and resistance among black women.

#### Conceptual framework of implementation

The CFIR has been widely adopted across a spectrum of research disciplines [52, 56–62]. It provides a useful lens through which to systematically examine key elements needed to ensure successful intervention adoption, implementation, fidelity, and sustainability. This innovative framework guides the study’s (1) research design; (2) exploration of the potential barriers and facilitators of implementation in a real world correctional setting; and (3) the qualitative components, measurement, and analytic plan for our mixed-methods aim. The specific domains of CFIR that have been applied to the implementation of E-WORTH include the following:

- *Outer setting*: We define *outer setting* as New York City Department of Probation and community corrections policies and procedures governing safety, facility access, and access to potential enrollees as a whole impacting recruitment techniques employed.
- *Inner setting*: Inner setting considerations include various organizational characteristics of community providers who serve women in community corrections settings that we partnered with to deliver E-WORTH. For the purposes of this article, we refer to this provider as E-WORTH’s nongovernmental organization (NGO) community provider. We also refer to the NGO community provider personnel that facilitate E-WORTH sessions as “facilitators.” Inner setting characteristics include networks and communication; culture, climate, and readiness for implementation; and policies and procedures related to participant engagement and support.
- *Individuals in the organization*: For E-WORTH, this construct includes study personnel at recruitment and implementation sites (e.g., probation, parole, NGO community), readiness to adopt the intervention, attitudes about technology (e.g., perception about how hard it is to use, how hard it is to administer to participants), and knowledge of and views about the intervention.
- *Intervention characteristics*: Intervention characteristics include features of the intervention itself (the use of a tablet, adaptability, complexity, design) that might compromise or facilitate implementation.
- *Implementation processes*: We define implementation processes as preplanning and early engagement activities, training, supervision strategies used, reflection and evaluation activities, fidelity monitoring, and hiring practices used.

**Fig. 1:**
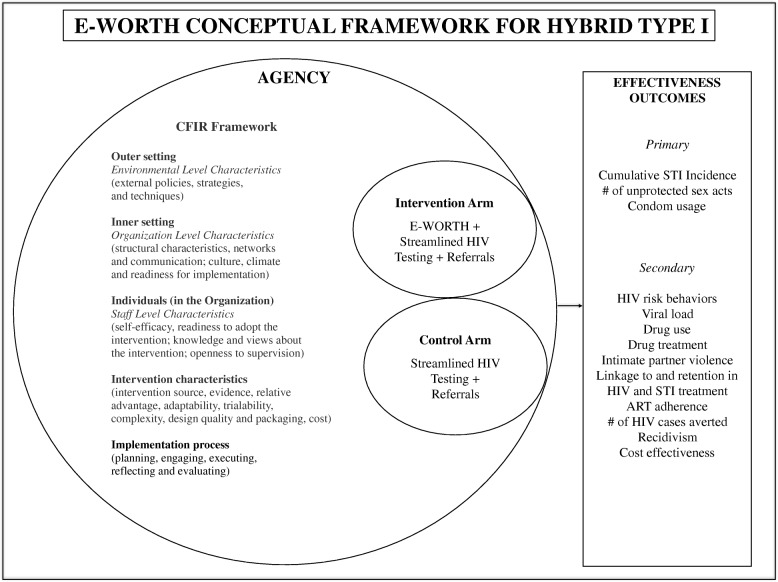
Conceptual framework for hybrid type I features the multilevel conceptual framework for the E-WORTH intervention and identifies applicable key Consolidated Framework for Implementation Research (CFIR) constructs [52] that enhance or diminish the delivery of Empowering African-American Women on the Road to Health (E-WORTH) and streamlined HIV testing in community corrections sites from early engagement through adoption. The figure also depicts the study’s intervention and control arms along with the primary and secondary outcomes. *ART* Antiretroviral therapy, *HIV* Human immunodeficiency virus, *STI* Sexually transmitted infection

Figure 1 features the multilevel conceptual framework for this intervention, which identifies applicable key CFIR constructs [52] that enhance or diminish the delivery of E-WORTH and streamlined HIV testing in community corrections sites from early engagement through adoption. Figure 1 also depicts the study’s intervention and control arms along with the primary and secondary outcomes.

Although we provide an overview of each domain, this trial primarily evaluates CFIR’s inner setting. In considering the feasibility of implementing E-WORTH in selected correctional settings, CFIR will aid us in answering critical questions such as the following:

1. What are the optimal strategies to employ during the preengagement phase to ensure sustainability through implementation and beyond?
2. What are the ways to best to supervise facilitators in correction settings?
3. What are the cultural considerations specific to community corrections settings that may facilitate or impede efforts to disseminate or scale up E-WORTH?

### Community-based participatory research

Guided by CBPR principles of shared and equitable decision making in all aspects of the research process, the investigative team convened a multistudy community advisory board during the preimplementation phase of the study [63]. The advisory board, formally referred to as the Community Collaborative Research Network (CCRN), is comprised of formerly incarcerated and other justice-involved men and women, New York City Department of Probation representatives, New York City Department of Health representatives, NGO community leadership personnel, and not-for-profit service providers from a variety of settings who serve justice-involved individuals. CCRN is guided by shared decision-making processes that are a hallmark of CBPR, and board members provide integral feedback on various aspects of E-WORTH’s implementation processes, including but not limited to participant outreach, recruitment, and retention.

CBPR principles also permeate other aspects of E-WORTH’s adaptation and implementation. Prior to the launch of the study, we held focus groups and piloted the study with black women with active or recent histories of justice involvement and substance use. Focus group and pilot participants helped to refine the study’s content, provide feedback on the newly Afrocentric “look and feel” of the adapted intervention tool, and tested the multimedia platform.

Also guided by the CBPR, the investigative team held regularly scheduled strategic planning meetings with NGO community facilitators and supervisory personnel during the preimplementation phase of the study to discuss implementation logistics. In addition, we began holding weekly strategic planning calls with NGO community personnel prior to the launch of the study to discuss more granular implementation details, including but not limited to marketing materials, staff training and supervision, and study protocols. Advisory board meetings, focus groups, and strategic planning meetings are being held continually through the conclusion of the study.

## Methods/design

### Study sites

Participants are recruited from multiple corrections organizations and community-based organizations in New York City recruitment sites, including the New York City probation and parole departments. We employ a variety of recruitment strategies when promoting E-WORTH at these locations. Columbia University project research assistants (RAs) directly approach potential enrollees and ask permission to talk to them about participation in the study. Project personnel also post intervention flyers and conduct presentations at each location.

The NGO community reentry provider with whom we entered into a service agreement contract is the largest nonprofit provider of services to community corrections populations in New York City. NGO community reentry sites in Manhattan and Queens serve as study sites. We selected this provider on the basis of their 50-year history of providing a constellation of services, including HIV testing and counseling, to justice-involved individuals. The NGO provider identified multiple part-time facilitators (equivalent to one full-time staff member) who met the following Columbia University criteria:

1. Must self-identify as African-American or black women;
2. Must have 2 years of experience in HIV, substance, and/or IPV care working with justice-involved populations; and
3. Must have a high school diploma or equivalent degree.

### Recruitment

**Fig. 2:**
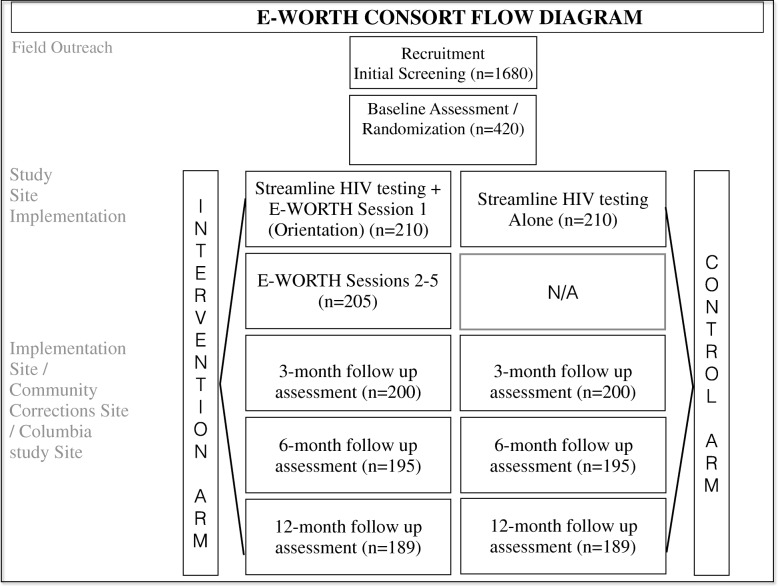
Empowering African-American Women on the Road to Health (E-WORTH) intervention flow. The figure depicts E-WORTH’s Consolidated Standards of Reporting Trials (CONSORT) diagram and provides a visual overview of all key stages in the intervention and control arms from outreach and screening through the 12-month follow-up assessment. *HIV* Human immunodeficiency virus

For the randomized effectiveness trial component of this study, we aim to recruit a total of 420 black women who use controlled drugs and are involved in the criminal justice system. We started enrolling participants in November 2015 and will complete enrollment in May 2018. Women assigned to either intervention condition (E-WORTH plus streamlined HIV testing or streamlined HIV testing alone) will receive all services provided by community NGO personnel. Women who test positive for HIV in either condition will receive HIV posttest counseling and linkage to treatment using the Centers for Disease Control and Prevention’s Guidelines for Counseling, Testing, and Referral. Figure 2, E-WORTH’s Consolidated Standards of Reporting Trials (CONSORT) diagram, provides a visual overview of all key stages in the intervention and control arms from outreach and screening through the 12-month follow-up assessment.

Trained Columbia University RAs distribute recruitment flyers that provide a telephone number and basic information regarding the intervention. Research staff also recruit in the waiting areas at corrections locations. If a woman expresses an interest in participating in the intervention, RAs invite her to be screened in a private room in an area away from community corrections (e.g., probation, parole) personnel and other corrections clients. The RA will obtain informed consent to be screened by reading the screening consent form to the participant and asking if she has any questions about the screening interview before obtaining her signature on the form. After obtaining informed consent, the RA will conduct a 10–15-minute screening interview to determine eligibility and willingness to participate.

Table 1 lists E-WORTH eligibility criteria. To be eligible, participants must meet all criteria.

**Table 1:** Eligibility criteria for effectiveness trial

Inclusion criteria	
Demographics	Assigned gender at birth (female) Identify as female 18 years of age or older Identify as African-American or black English-speaking If participant is pregnant, she is less than 7 months pregnant Has an address to receive mail Lives in one of the five boroughs of New York City
Criminal justice involvement	Supervised by a criminal justice entity, such as probation, parole, or alternative-to-incarceration program in the past 90 days
Sexual risk behavior	Reports engaging in unprotected vaginal or anal sex with a male partner in the past 90 days
Substance use risk	Reports any illicit drug use or binge drinking or enrolled in alcohol or drug treatment in the past 6 months
Other substance use or sexual risk	Reports at least one of the following outside risks: 1. Had more than one male partner in the past year 2. Injected drugs in the past year 3. Was ever diagnosed with human immunodeficiency virus (HIV), herpes, or genital warts 4. Was diagnosed with a sexually transmitted infection (STI), including gonorrhea, syphilis, chlamydia, trichomoniasis, pubic lice, etc., in the past year or 5. Has had sex with a male partner in the past year who she knows or suspects of having at least one of the following: a. Partner is HIV-positive, positive for hepatitis C virus, or has herpes or genital warts b. Partner has had any other STI in the past year c. Partner has had sex with another person

**Fig. 3:**
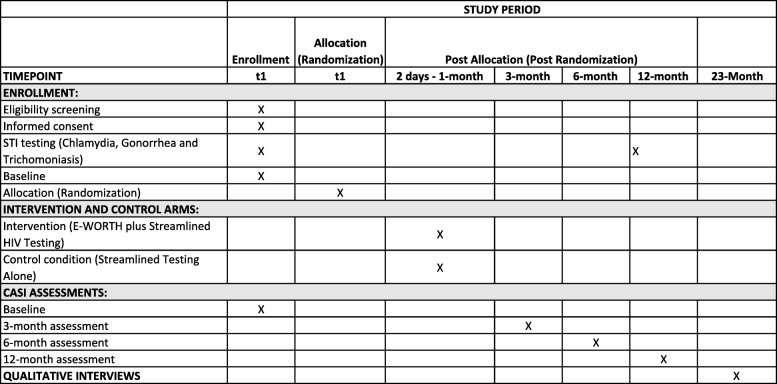
Empowering African-American Women on the Road to Health (E-WORTH) participant Standard Protocol Items: Recommendations for Interventional Trials (SPIRIT) figure. Provides an overview of the schedule and time commitment related to the intervention and control arms

Participants receive $5 for completing an initial screening. If they meet eligibility criteria for the intervention and are willing to participate, an enrollment meeting will be held at a community NGO provider site. Participants receive $55 for the baseline interview and $55, $60, and $65 respectively, for the 3-, 6-, and 12-month follow-up appointments. Compensation amounts include HIV and STI testing and cover all study-related transportation costs. Follow-up assessments are administered at a community NGO provider location, Columbia University’s community research site, or at a community corrections location (i.e., the probation or parole office from which the participant was originally recruited) (Additional file 1). Figure 3 depicts E-WORTH’s Standard Protocol Items: Recommendations for Interventional Trials (SPIRIT) figure and provides an overview of the schedule and time commitment related to the intervention and control arms.

The enrollment meeting consists of the following steps:

1. A computerized baseline assessment;
2. Testing for chlamydia, gonorrhea, and trichomoniasis;
3. Assignment to an intervention condition;
4. For those assigned to the intervention arm, completion of a brief introductory session of the E-WORTH intervention (session 1); and
5. A streamlined HIV test administered during session 1.

Informed consent will again be obtained, and a confirmatory screening also will be conducted for all participants whose enrollment appointment occurs within more than 24 hours of the date they were screened. The study consent form indicates that research staff may also contact participants at a future date to determine if they may be interested in participating in additional studies. The consent form also notes that participation in future studies is completely voluntary. Participants are given a copy of the consent form and, upon request, all additional relevant study information (e.g., copies of the research protocol). Participants assigned to either intervention condition also receive a comprehensive referral manual tailored for drug-using, justice-involved black women. The study’s manual lists health, mental health, employment, general services, and treatment resources throughout New York City.

Participants are enrolled at the community NGO provider site of their choice (typically one close to their place of residence) by Columbia University RAs. Appointments are held on the day participants are screened or at the potential participant’s earliest convenience. Columbia University personnel complete steps 1 and 2 of the enrollment meeting; community NGO staff complete the remaining steps. Appointments are held in the computer laboratory or conference room areas of the NGO community reentry location.

Assignment into an intervention arm takes place on the day and at the time of the enrollment meeting. If a participant indicates that they are not able to stay for the portion of the enrollment appointment in which they are assigned to an intervention arm, steps 3–5 are not completed. The participant will instead be asked to return on the very next business day they are available. Participants must be assigned to an intervention arm within 45 days of their initial visit. If the participant is not available within this time frame, she must again complete the computerized baseline assessment and then complete session 1 and be tested for HIV.

### Randomization

Consistent with an intention-to-treat approach for data analyses, participants are considered enrolled in the trial only after they are assigned to an intervention arm during their enrollment appointment. Assignment to an intervention condition occurs at both community NGO locations concurrently but independently; that is, the numbers and timing of randomization and launching of intervention groups in one site does not affect assignments or launching in the other intervention location.

We balance first by intervention condition and second by the community NGO site at which the enrollment interview is completed. To ensure that a comparable number of participants enter the intervention and control conditions at a given study site, we use a hybrid randomization approach in which participants are both randomly and “purposefully” assigned to an intervention condition. Allocation sequences are automatically generated using an Excel spreadsheet (Microsoft, Redmond, WA, USA) managed by the study’s project director. Periods of randomization are automatically followed by periods of purposeful assignments. To eliminate bias during periods of “purposeful” assignments, we adhere to fixed randomization and assignment windows governed both by calendar days and by the total time it takes enroll the requisite number of women to launch a new intervention group.

### Intervention condition: E-WORTH and streamlined HIV testing

Consisting of the individual introduction to E-WORTH and streamlined HIV testing session followed by four 90-minute group sessions, E-WORTH optimizes group and individual modalities in a hybrid intervention design. Group sessions feature an “in-room” facilitator who provides support and guidance through group opening and closing activities. In addition, E-WORTH employs computerized interactive exercises and video testimonials of women through the aid of a tablet-based “online” narrator that leads participants through computerized self-paced modules.

**Fig. 4:**
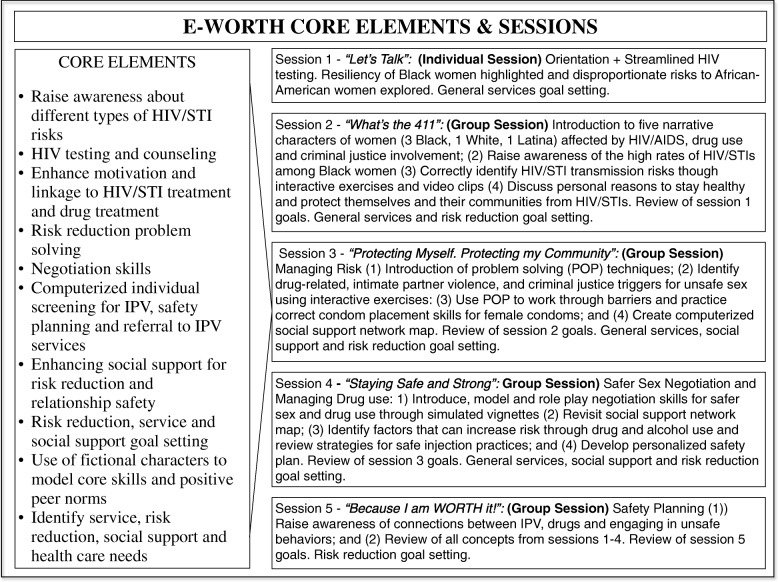
Empowering African-American Women on the Road to Health (E-WORTH) core elements and sessions. The figure provides an overview of each intervention session. It also provides an overview of the core elements (components) of the intervention. *HIV* Human immunodeficiency virus, *IPV* Intimate partner violence, *STI* Sexually transmitted infection

Figure 4 provides an overview of each intervention session. It also provides an overview of the core elements (components) of the intervention. E-WORTH’s core components (*see* Fig. 3) include raising awareness about different types of HIV/STI risks, promoting HIV testing and counseling, enhancing motivation and linkage to HIV/STI treatment and drug treatment, and teaching risk reduction problem solving. Participants also learn negotiation skills and establish risk reduction, general service, and social support goals. Embedded in the multimedia platform are tools for IPV, safety planning, and referral to IPV services. The computerized tool also aids in social support building for risk reduction and relationship safety, and it features fictional characters that model core skills and positive peer norms. E-WORTH’s core elements are designed to raise participants’ awareness about different types of HIV/STI risks and increase their motivation for testing, counseling, and treatment. E-WORTH’s core elements are designed to raise participants’ awareness about different types of HIV/STI and IPV risks and increase their motivation for testing, counseling, and treatment. E-WORTH also aims to decrease the incidence of IPV at follow-up assessments.

E-WORTH group sessions are delivered in a traditional paper-based group setting by a trained NGO community reentry facilitator, but the sessions also have computerized self-paced modules that individual members complete on tablets. This hybrid group modality, in which about half the session activities are delivered as computerized self-paced modules, was found to be efficacious in reducing unprotected sex compared with an attentional Wellness Promotion control group [3, 64]. E-WORTH’s innovative multimedia components include the use of narratives and videos with fictional characters who portray different life stories of predominantly black women affected by HIV/AIDS, STIs, drug use, and criminal justice system involvement. The intervention also provides instruction and demonstration of core skills (e.g., safer sex negotiation and problem-solving skills, technical condom use skills) through the use of culturally congruent role models. Participants also complete individual computerized exercises and logs designed to enhance recall of core knowledge and to provide a confidential space for participants to track their individual progress in reducing risky behaviors and achieving risk reduction goals.

### Facilitator training, technical assistance, adherence, and supervision

All NGO community facilitators receive a 4-day training led by Columbia University personnel on how to deliver the E-WORTH intervention. Facilitation training focuses on developing competency on the core components of the E-WORTH intervention using Motivational Interviewing (MI) techniques. MI is an evidence-based, participant-centered treatment technique with proven efficacy in people with mental health and substance use disorders [65]. MI is a strengths-based approach that supports and promotes client self-efficacy, expresses empathy, and motivates clients to change self-destructive behaviors.

Columbia University personnel meet with NGO community provider facilitation and supervisory personnel on an as-needed basis to coordinate study implementation activities and delivery. The NGO provider also assigns one manager at each study site to provide integral logistical support in navigating implementation challenges as they arise. Challenges include but are not limited to participant access to community NGO sites, technological failures and Wi-Fi access issues, and staff scheduling emergencies. Columbia University and NGO supervising staff are also on-call to assist facilitators in the event of clinical emergencies that may emerge with group participants.

E-WORTH’s multimedia platform records attendance data for each intervention session and information on number and general type of referrals to services using participant identification numbers. NGO community provider facilitation personnel also complete a session adherence form for each session that assesses the extent to which core elements and activities of the session are covered and the quality of delivery of each activity using a rating scale. In addition, the computerized multimedia E-WORTH tool will generate data on whether participants completed each session activity and the amount of time spent on each activity.

NGO facilitators also receive biweekly group supervision by conference call with Columbia University’s E-WORTH project director or a member of the study investigative team. During supervision calls, facilitators review session adherence forms, discuss challenging situations that arise, and develop strategies for responding to such challenges in the future. E-WORTH’s project director is also available for technical assistance calls as needed related to challenges with implementing computerized modules or specific requests for individual supervision from facilitators. We collect data on the nature and type of technical assistance requests and clinical emergencies that occur, as well as on the attendance of participants and content covered in clinical supervision calls. Standard services at the NGO community reentry provider site are offered to participants randomized to both intervention conditions. Core programming includes social services, HIV testing and counseling, on-site and off-site substance abuse and mental health treatment referrals, and educational and vocational services.

### Control condition: streamlined HIV testing

Community NGO facilitators deliver a 5-minute HIV testing information session in a private space to participants assigned to the control condition. During the information session, facilitators describe the rapid testing procedure as well as the timing for and meaning of test results, and they explain the window period during which an antibody test might be negative. Facilitators briefly describe what HIV infection is and give participants HIV testing kit material, which provides an overview of transmission risks and effective strategies for reducing risk. This streamlined HIV testing intervention is consistent with New York State law and does not require risk reduction counseling at the time of HIV testing. The streamlined HIV testing alone condition has been tested and found to be effective in two large-scale multisite studies conducted in the National Institute on Drug Abuse (NIDA) Clinical Trials Network [47, 48].

### Retention

RAs are trained to engage participants in identifying and overcoming barriers to attendance at follow-up sessions. We use a variety of retention strategies, including administering a detailed locator package at baseline, assigning cases to RAs to track from baseline to the 12-month follow-up, and updating locator data at every assessment. Because the study involves participants who are completing community corrections sentences, there is a likelihood that some participants may become incarcerated. If that does occur, the study’s adverse events protocol will be followed, and the participant’s involvement in the study will be discontinued. In addition, during the informed consent meeting, potential participants are apprised that they may withdraw from the study at any time. In the event that a participant voluntarily withdraws from the study or the investigator decides to discontinue a participant owing to an adverse event, no additional assessment data are collected.

### Criteria for discontinuing or modifying intervention

The study protocol provides for ancillary and posttrial care and compensation for participants who are deemed to have suffered. Data on adverse events, including those that are psychological in nature, are systematically collected during the trial and reported to the principal investigative team and the study’s institutional review board (IRB) in order to ensure appropriate management of the adverse events as well as to have full documentation of the events for all conditions. Adverse events are also reported to the study’s IRB and data and safety monitoring board (DSMB). The DSMB consists of four expert members in the field who are experienced with HIV treatment, substance abuse treatment, and vulnerable populations, all of whom have no conflict of interest with regard to the study. The DSMB oversees and provides advice to the principal investigative team on the continued scientific integrity and safety of the implementation of the trial. The DSMB reviews the status of recruitment, retention, successes, barriers and challenges of the implementation of the trial, adverse events, and the reporting procedures of adverse events. Two interim analyses will be conducted in addition to the planned “final” analyses at the conclusion of the proposed study. The DSMB will review findings from the interim analysis and determine whether the study needs to be terminated for safety reasons. Data will be sent to the DSMB 1 week prior to the meeting. The DSMB meets twice per year. The meetings are face-to-face, and minutes taken at the meeting are archived.

Interim analyses use complete data over the longest follow-up duration (i.e., the 12-month assessment time point). The principal investigators and coinvestigators will be unblinded to the interim analyses, but the study staff will be blinded. The interim analyses will be conducted by the statistician on the study, and the results will be reported to the principal investigators. With respect to efficacy, stopping rules following an interim analysis focus on demonstrable benefit in the sample and based on data accrued to date.

### Monitoring fidelity of implementation: process measures and quality assurance procedures

We employ several strategies endorsed by the Centers for Disease Control and Prevention Capacity Building Branch for the dissemination of evidence-based interventions to enhance fidelity and monitor “intervention drift.” Our strategies include the following:

1. Providing standardized training and ongoing supervision for NGO community reentry facilitators who deliver both conditions to enhance competency;

2. Scheduling regular site visits to meet with NGO community reentry partners to discuss challenges and assess quality and fidelity of implementing both conditions;

3. Collecting data on number and type of technical assistance requests;

4. Monitoring sessions for both conditions by listening to a randomly selected percentage of audio-recorded sessions for quality assurance to rate how closely facilitators adhere to the intervention protocol and the overall clinical quality of delivery each session activity; and

5. Regularly reviewing E-WORTH’s fidelity assessment tool, which records how much time is spent on the activity and records individual participant responses to risk ratings and goal setting.

The use of E-WORTH’s fidelity assessment tool along with the collection of process measures also allows us to rigorously monitor the fidelity of implementing E-WORTH plus streamlined HIV testing and streamlined HIV testing alone conditions.

### Procedures to handle treatment contamination

Because participants are randomized at NGO community reentry provider intervention sites and may have some interaction with other potential enrollees, there is a risk of contamination between intervention and control arms. We employ a variety of quality assurance measures to minimize or avert contamination risks between treatment conditions. All recruitment and randomization activities are completed under the supervision of Columbia University’s leadership personnel, and RAs are blinded to the randomization process. In addition, all enrollment meetings and E-WORTH intervention sessions are held in private areas to minimize contact between participants randomized to intervention and control arms. Research staff are also trained to identify and investigate any detected instance(s) of contamination between intervention arms and engage in immediate corrective action.

### Measures

Participants complete computerized assessments at baseline and at the 3-, 6-, and 12-month follow-up marks. Columbia University RAs schedule all follow-up appointments and meet with participants at intervention sites. Table 2 provides a description of E-WORTH’s effectiveness trial measures.

**Table 2:** Effectiveness trial measures

Variable type	Construct	Description	Timeline
Primary outcome	Reduced incidence of biologically confirmed STIs (i.e., chlamydia, gonorrhea, and trichomoniasis)	Biological assay for infection by *Chlamydia trachomatis*, *Neisseria gonorrhoeae*, and *Trichomonas vaginalis*	Baseline, 12-month follow-up
Secondary outcomes	HIV risk behaviors (number of unprotected sex acts; self-reported condom use)	Measured using NIDA’s Seek, Test, Treat and Retain for Vulnerable Populations: Data Harmonization Measure Sexual risk behavior items are based on the Women’s Health CoOp Baseline Questionnaire	Baseline, 3-month, 6-month, 12-month follow-up
	Viral load	Written confirmation of viral load information for HIV-positive participants provided by participant’s medical provider	Baseline, 3-month, 6-month, 12-month follow-up
	Drug use	Measured using NIDA’s Seek, Test, Treat and Retain for Vulnerable Populations Data Harmonization Measure Injection risk behavior items are taken from the STTR Criminal Justice instrument	Baseline, 3-month, 6-month, 12-month follow-up
	Use of drug treatment	Single-item question that inquires about participant’s current legal criminal justice status Participants are asked to select all responses that apply, including mandated drug treatment court sentence.	Baseline, 3-month, 6-month, 12-month follow-up
	Intimate partner violence	IPV was assessed using three subscales from the Revised Conflict Tactics Scale (CTS2) (physical, sexual, and injury-related) [71].	Baseline, 3-month, 6-month, 12-month follow-up
		We also used the four-item Jellinek Inventory for assessing Partner Violence [72].	Baseline
		Last, we assessed IPV using a two-item self-efficacy inventory that gauges participants’ attempts to make or update a safety plan, or obtain either an order of protection or a restraining order.	Baseline, 3-month, 6-month, 12-month follow-up
	Linkage to and retention in HIV and STI treatment	We assessed linkage to and retention in HIV and STI treatment using self-reported items gauging the receipt of medical care for HIV.	Baseline, 3-month, 6-month, 12-month follow-up
	ART adherence	We also assessed linkage to and retention in HIV and STI treatment using self-reported items inquiring about the use of ART	Baseline, 3-month, 6-month, 12-month follow-up
	Criminal justice involvement and recidivism	Self-reported items assessing number of times stopped, detained, arrested, and convicted of a crime during assessment time frames.	Baseline, 3-month, 6-month, 12-month follow-up
Moderators	Sociodemographics	Self-reported data collected on age, gender, race/ethnicity, education, income, and marital status.	Baseline
	Psychosocial characteristics	Depression is assessed using the four-item Center for Epidemiologic Studies Depression Scale.	Baseline, 3-month, 6-month, 12-month follow-up
		Posttraumatic stress experienced in the past month was assessed using the PCL-C [73], a standardized self-report rating 17-item scale that corresponds to the key symptoms of PTSD.	Baseline, 12-month follow-up
		We use the Stressful Life Events Screening Questionnaire to assess lifetime exposure to traumatic events [74].	Baseline
	CFIR construct: outer setting (Environmental-level characteristics)	Questions that focus on the external environment of all recruitment and intervention locations (i.e., probation sites, NGO community reentry provider locations, recruitment locations, strategies and techniques). Assessed using structured qualitative interviews and computerized surveys administered to NGO community reentry facilitators at repeated time points.	Ongoing
	CFIR construct: inner setting (organization-level characteristics)	Items inquiring about the feasibility of implementing the intervention in all identified locations; the structural characteristics, networks and communication, culture, climate and readiness for implementation of the implementation organization, recruitment sites and probation locations. Also assessed using structured qualitative interviews and computerized surveys administered to NGO community reentry facilitators at repeated time points.	Baseline, repeated assessments
	CFIR construct: individuals setting (staff-level characteristics)	Self-reported questions that capture NGO community reentry facilitators’ readiness to adopt the intervention; knowledge and beliefs about the intervention that might influence adoption; self-efficacy regarding ability to deliver the intervention; individual identification with organization; attitudes about technology (e.g., perception about how hard it is to use, how hard to administer to participants); and other personal attributes.	Baseline, repeated assessments
	CFIR construct: intervention characteristics	Determined using self-reported survey questions, this construct examines: community reentry facilitators’ and organizational providers’ attitudes towards the intervention; and features of the intervention itself (the use of a tablet to deliver the intervention, adaptability, complexity, design) that might compromise or facilitate implementation.	Baseline, repeated assessments
	CFIR construct: process	These self-reported inventory items captures and processes activities associated with preplanning and early engagement and implementation, supervision of intervention, and evaluation of the intervention.	Baseline, repeated assessments

*Abbreviations: ART* Antiretroviral therapy, *CFIR* Consolidated Framework for Implementation Research, *HIV* Human immunodeficiency virus, *IPV* Intimate partner violence, *NGO* Nongovernmental organization, *NIDA* National Institute on Drug Abuse, *PCL-C* Posttraumatic Stress Disorder Checklist–Civilian version, *PTSD* Posttraumatic stress disorder, *STI* Sexually transmitted infection, *STTR* Seek, Test, Treat and Retain

### Power analysis

For E-WORTH’s primary outcome, we used intraclass correlation estimates based on previous WORTH trials, and variance inflation factors were calculated to generate effective sample sizes used in G*Power for the effectiveness aim. For the target sample size calculation, we selected cumulative STI incidence for our power analysis, given that it is our primary outcome and that it had the lowest power among the other outcome variables, and we assume a 50% reduction (on a background cumulative incidence of 20.5%) to be clinically meaningful to detect via Poisson regression. For behavioral outcomes, projected means and SDs allow for effect size *f*_2_ to be calculated and used in G*Power [66]. Results for primary outcomes indicate that 80% power is achieved for the rate-limiting outcome (i.e., cumulative STI incidence) with an effective sample size of 372 participants, translating to a sample size of 378 participants after accounting for intraclass estimates. We then increased the starting sample size to 420 to safeguard against attrition.

### Cost-effectiveness

In response to the need for greater transparency regarding the allocation of limited federal funding for scientific research, cost-effectiveness analytical techniques have increasingly been used in evidenced-based research [67, 68]. Despite this fact, scientific guidelines for deciding how best to allocate scarce resources to promote uptake of cost-efficient HIV services in correctional settings have yet to be developed. Although it is true that cost-effectiveness is only one of many considerations when disseminating research into practice settings, the importance of cost-effectiveness in correctional settings marked by funding constraints and limited human resources cannot be minimized. E-WORTH also addresses this gap. We aim to prospectively evaluate costs of delivering E-WORTH and streamlined HIV testing by generating comparative cost-effectiveness data that will enable policy makers and program administrators to identify the most appropriate HIV/STI interventions for criminal justice settings. The technological components of E-WORTH also have considerable cost and sustainability implications because E-WORTH requires a lower degree of professional education, training, and supervision to deliver.

We define cost-effectiveness as the incremental cost of delivering E-WORTH divided by two intervention outcomes: (1) differences in biologically observed STIs (chlamydia, gonorrhea, and trichomoniasis) and (2) differences in biologically confirmed HIV cases between the E-WORTH intervention arm and streamlined HIV testing and the streamlined HIV testing alone arm. In addition, we will explore cost-related considerations associated with implementing E-WORTH in a real-world setting.

We will collect intervention-specific cost data from a variety of sources to calculate the incremental cost of delivering E-WORTH. Cost data include the following: (1) capital costs (e.g., space/rent information provided by the NGO community reentry provider sites); (2) salary/personnel/labor information (e.g., total time spent on intervention preparation and delivery based on self-reported time sheets); (3) cost spent on study participants: goods, supplies, travel reimbursement, and test kits; and (4) research-related intervention costs (e.g., staff time spent supervising intervention facilitators).

### Data analyses

#### Effectiveness trial: aims 1 and 2

The analytic approaches used for E-WORTH are built upon the investigative team’s prior HIV prevention randomized controlled trials, which involve an intention-to-treat approach, handling missing data using multiple imputation, and conducting sensitivity analyses to quantify robustness of findings on the basis of models and their assumptions. Descriptive statistics will characterize the sample and measurement distributions to ensure proper application of multivariate methods. We will use multiple imputation to handle missing data. Preliminary analyses include bivariate analyses to identify unadjusted associations among variables. Variables significantly associated with outcome variables and attrition, as well as significantly different across intervention arms, will be included as covariates during hypothesis testing. We have also constructed a qualitative dataset that will aid in data analysis of key themes that emerge.

#### Cost-effectiveness: aim 3

To better understand cost-related considerations associated with implementing E-WORTH in a real-world setting, qualitative interviews also will be conducted with implementation facilitation and supervisory personnel focusing on staff turnover, training, and staff supervision needs. Interview findings will aid us in determining how best to scale up E-WORTH after the clinical trial has been completed. We anticipate substantially lower statistical power for observed HIV seroconversions than for STIs. As a result, for the HIV outcome “denominator” in the cost-effectiveness calculation, we will use an “HIV infections averted” estimate using data from the trial on HIV risk behavior and a model of HIV transmission used in HIV epidemiology and cost-effectiveness literature [69, 70]. Sensitivity analyses will also be performed (e.g., using highest and lowest reported values for model parameters reported among different studies) to ascertain the extent to which cost-effectiveness conclusions are robust to parameter assumptions.

#### Implementation: aim 4

**Fig. 5:**
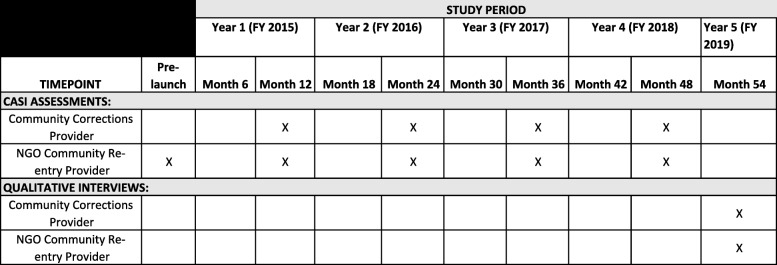
Empowering African-American Women on the Road to Health (E-WORTH) project Standard Protocol Items: Recommendations for Interventional Trials (SPIRIT) figure. Provider study-related items. The figure provides an overview of each study time point related to provider assessments and interviews. *CASI* Computer-assisted self-interview, *NGO* Nongovernmental organization

Guided by the CFIR, we will collect quantitative and qualitative data to identify multilevel factors and processes that enhance or diminish the fidelity of implementation, such as perceptions about the benefits and disadvantages of delivering the intervention at their site, perceptions of clients’ reactions to the intervention, perceptions of the organizational climate of their probation site, and their attitudes toward criminal justice as a whole and HIV/STI interventions targeting substance-using women. We will administer structured, computerized assessments to NGO community reentry providers, probation and parole providers, and various community corrections personnel. Survey questions will include items related to sociodemographics and professional training of staff and providers, attitudes toward the E-WORTH and streamlined HIV testing and the streamlined HIV testing alone conditions, level of self-efficacy in delivering interventions, organizational readiness to change, and organizational climate. From an implementation perspective, attitudes about technology (e.g., perception about how difficult it is to use, how complex it is to administer to participants, how expensive might it be for the community-based organization (CBO) to operate) will have an impact on an organization’s choice whether to implement it. Figure 5 provides an overview of each study time point related to provider assessments and interviews.

### Integration of qualitative and quantitative findings

As a final step, we will integrate all qualitative and quantitative results using the E-WORTH multilevel CFIR informed conceptual framework to triangulate findings and provide a full picture of mechanisms that influence the fidelity of implementation and the effectiveness of the intervention. We will conduct multilevel, parallel mixed data analysis to isolate findings that emerge from each method and to clarify its transferability. We will separately analyze quantitative and qualitative components and generate inferences and conclusions in each aim. We will then make metainferences by integrating inferences obtained from all study strands [67]. Because parallel analysis can lead to convergent or divergent metainferences, we will use these as opportunities to clarify applicability of theory, methodologies, and interpretations to broader contexts.

### Data management and confidentiality of study data

Data management activities and procedures use electronic data management systems designed to enhance the efficiency, security, and integrity of study data. Data collected using computer-assisted self-interviews (CASIs) during baseline and follow-up assessments are automatically recorded/stored by the computer. As respondents move through computerized assessments, responses are encrypted for transit between the respondent’s browser and the study’s server using SSL and 128-bit encryption.

All qualitative interviews will be audio-recorded and transcribed. We will construct a qualitative dataset that will include identification of each transcript as well as inclusion of relevant demographic data by provider/probation staff and by probation site that will aid in data analysis of key themes that emerge. A preliminary set of analytic coding categories (closed codes) will be assembled on the basis of concepts, ideas, themes, and patterns that characterize multilevel theory-driven factors or processes described above. This set of codes will be continually updated through a process of contrast and comparison. The initial or open-coded data are then organized under the analytic category list. Digital recordings of in-depth qualitative interviews, in-depth qualitative interview transcripts, and survey data from the CASI interview will be assigned a study identifier code. Digital audio recordings are also uploaded for quality assurance protocols. Only the principal investigators and the study’s project director have password-protected access to the encrypted file linking names of participants and their study identifier codes. Participants will be assigned a unique identifier code that is different from their study identifier code to log into the multimedia platform. The file linking the multimedia platform identifier and the study identifier will be accessible only to the principal investigator and the study’s project director. The multimedia platform will only collect use data regarding the resources in the program. The program will not collect personally identifying information. Use data that are collected by the multimedia platform will be password-protected and accessible only to the principal investigator and the study’s project director.

Community corrections and NGO community reentry providers will not seek access to any personally identifying study data provided by individuals or community corrections employees collected by Columbia University. Columbia University will provide community corrections and NGO community reentry personnel with aggregated findings in a final project report at the conclusion of the study. The identities of study participants and employees will be masked in this report and any report produced by Columbia University such that it will not be possible to identify individuals using information provided in the report.

## Discussion

The intertwined risks of HIV/STI transmission and IPV for black women who use drugs and are undergoing community correction remain extremely high. Adapting evidence-based HIV/STI and IPV prevention interventions such as the original WORTH intervention for this population holds tremendous promise for addressing this public health crisis. To date, there have been no HIV/STI and IPV prevention interventions specifically directed toward helping black women in community corrections gain more knowledge about their HIV/STI status and become more resilient and self-protective of their health and safety.

By targeting drug-using black women specifically, the subset of the justice-involved female population at the highest risk for HIV/STIs and IPV, this study advances understanding of culturally tailored strategies to enhance the protective practices of this target population of black women. Community correction settings provide an untapped venue for reaching this highly vulnerable but difficult-to-engage population. Nevertheless, strategies for implementing HIV/STI and IPV interventions in organizational settings that serve this population are as yet poorly understood.

This hybrid type I design protocol addresses this gap. Lessons learned from the experiences of correctional personnel in delivering this intervention will bridge the informational gap between research and implementation science in this context. Key areas of focus include community corrections provider attitudes toward E-WORTH. Data gathered will explore the ways in which features of the intervention itself (adaptability, complexity, design) might impede or facilitate implementation. E-WORTH will also offer useful insight regarding optimal strategies to use when recruiting, training, and supervising providers in correctional settings who deliver interventions of this type. In addition, E-WORTH will provide invaluable information on what is required organizationally to run a culturally tailored group intervention for black women who use drugs in a real-world correctional setting.

Through its innovative design in implementing a group-based modality that includes a computerized self-paced tool in a community correction setting, this study provides a unique lens through which to understand the best strategies to employ this hybrid modality with technology in nonprofit settings as a whole and specifically in settings serving justice-involved populations. The use of fictionalized characters and simulated video vignettes to portray different life stories of black women affected by HIV/STIs, IPV drug use, and criminal justice involvement highlights E-WORTH’s innovative use of technology to deliver culturally congruent messaging tailored specifically for this population. E-WORTH’s online narrator also offers important insight regarding ways to creatively use technology to extend the reach of traditional “in the room” facilitators in delivering interventions in correctional settings. It also has important implications for expanding the use of technology to extend the reach of criminal justice providers in engaging hard-to-reach justice-involved women who remain at risk of HIV/STIs. The partially self-paced format points in the direction of either the need for less facilitator involvement or the possibility of administering the intervention to larger numbers of participants at one time, which has an impact on cost.

The analytic methods we employ in rigorously evaluating effectiveness and cost-effectiveness meet a NIDA funding priority while providing valuable data regarding the true cost of implementing studies such as E-WORTH in criminal justice settings. This is of particular importance, given the long-term financial and public health implications associated with engaging this high-risk but difficult-to-reach population. Although the value of averted HIV/STI cases cannot be fully quantified, cost-effectiveness calculations provide a useful and objective lens for evaluating the incremental cost of the E-WORTH intervention delivery by outcome. This too meets an important need in light of the current climate of budgetary cuts for scientific research and the limited availability of resources in nonprofit settings.

This study is designed to yield important data on various innovative approaches we employ in Project E-WORTH that have not been used, to our knowledge, in implementation science research. For example, we will examine the process by which we have embedded services tailored specifically for black women into organizational settings that provide services to men and women from all racial and ethnic backgrounds. We will identify the organizational benefits and challenges encountered in targeting E-WORTH for black women specifically and the potential impact on organizational climates. We will also identify effective strategies to use when recruiting intervention participants from various correctional settings. Additionally, we will assess the effectiveness of the CBPR strategies we used when introducing HIV and STI risk reduction and IPV prevention services into correctional settings.

## Trial status

Trial recruitment started in November 2015. Recruitment is ongoing. The project is scheduled to end on June 30, 2019.

## Additional file

Additional file 1: SPIRIT 2013 checklist: recommended items to address in a clinical trial protocol and related documents. (DOC 138 kb)

## Abbreviations

ART: Antiretroviral therapy
CASI: Computer-assisted self-interview
CBPR: Community-based participatory research
CCRN: Community Collaborative Research Network
CFIR: Consolidated Framework for Implementation Research
CONSORT: Consolidated Standards of Reporting Trials
DSMB: Data and safety monitoring board
E-WORTH: Empowering African-American Women on the Road to Health
HIV: Human immunodeficiency virus
IPV: Intimate partner violence
IRB: Institutional review board
MI: Motivational Interviewing
NGO: Nongovernmental organization
NIDA: National Institute on Drug Abuse
PCL-C: Posttraumatic Stress Disorder Checklist–Civilian version
PTSD: Posttraumatic stress disorder
RA: Research assistant
SPIRIT: Standard Protocol Items: Recommendations for Interventional Trials
STI: Sexually transmitted infection
STTR: Seek, Test, Treat and Retain
WORTH: Women on the Road to Health

## References

[1] Altice FL (2005). Correlates of HIV infection among incarcerated women: implications for improving detection of HIV infection. J Urban Health.

[2] Inciardi JA (2008). HIV and other infectious diseases among drug-involved offenders. J Psychoactive Drugs.

[3] El-Bassel N (2014). Efficacy of a group-based multimedia HIV prevention intervention for drug-involved women under community supervision: project WORTH. PLoS One.

[4] Rowell-Cunsolo TL, El-Bassel N, Hart CL (2016). Black Americans and incarceration: a neglected public health opportunity for HIV risk reduction. J Health Care Poor Underserved.

[5] El-Bassel N (2017). Women in community corrections in New York City: HIV infection and risks. Int J STD AIDS.

[6] Pelligrino N (2017). Incarcerated black women in the southern USA: a narrative review of STI and HIV risk and implications for future public health research, practice, and policy. J Racial Ethn Health Disparities.

[7] Centers for Disease Control and Prevention (CDC), Division of STD Prevention, National Center for HIV/AIDS, Viral Hepatitis, STD, and TB Prevention. STDs in racial and ethnic minorities. https://www.cdc.gov/std/stats15/minorities.htm. Accessed 23 Aug 2017.

[8] Centers for Disease Control and Prevention (CDC), Division of STD Prevention, National Center for HIV/AIDS, Viral Hepatitis, STD, and TB Prevention. HIV and African Americans. https://www.cdc.gov/hiv/pdf/group/racialethnic/africanamericans/cdc-hiv-africanamericans.pdf. Accessed 23 Aug 2017.

[9] Centers for Disease Control and Prevention (CDC), Division of STD Prevention, National Center for HIV/AIDS, Viral Hepatitis, STD, and TB Prevention. HIV and women. https://www.cdc.gov/hiv/pdf/group/gender/women/cdc-hiv-women.pdf. Accessed 23 Aug 2017.

[10] American Civil Liberties Union (ACLU). Facts about the over-incarceration of women in the United States. https://www.aclu.org/other/facts-about-over-incarceration-women-united-states. Accessed 22 Aug 2017.

[11] The Sentencing Project. Women in the criminal justice system: briefing sheets. May 1, 2007. https://www.sentencingproject.org/publications/women-in-the-criminal-justice-system-briefing-sheets/. Accessed 23 Aug 2017.

[12] The Sentencing Project. Incarcerated women and girls. http://www.sentencingproject.org/wp-content/uploads/2016/02/Incarcerated-Women-and-Girls.pdf. Accessed 23 Aug 2017.

[13] Minton TD, Golinelli D (2014). Jail inmates at midyear 2013 - statistical tables.

[14] Koch DW, Lee J, Lee K (2016). Coloring the war on drugs: arrest disparities in black, brown, and white. Race Soc Probl.

[15] Justice Center, Council of State Governments (2016). Building trust and legitimacy within community corrections.

[16] Vera Institute of Justice (2013). The potential of community corrections to improve safety and reduce incarceration.

[17] Belenko S (2004). HIV risk behaviors, knowledge, and prevention education among offenders under community supervision: a hidden risk group. AIDS Educ Prev.

[18] Adimora AA, Schoenbach VJ (2005). Social context, sexual networks, and racial disparities in rates of sexually transmitted infections. J Infect Dis.

[19] Adimora AA, Schoenbach VJ, Doherty IA (2006). HIV and African Americans in the southern United States: sexual networks and social context. Sex Transm Dis.

[20] Adimora AA, Schoenbach VJ, Floris-Moore MA (2009). Ending the epidemic of heterosexual HIV transmission among African Americans. Am J Prev Med.

[21] Gilbert L (2016). Efficacy of a computerized intervention on HIV and intimate partner violence among substance-using women in community corrections: a randomized controlled trial. Am J Public Health.

[22] Beckwith CG (2010). Opportunities to diagnose, treat, and prevent HIV in the criminal justice system. J Acquir Immune Defic Syndr.

[23] Kramer K, Comfort M (2011). Considerations in HIV prevention for women affected by the criminal justice system. Womens Health Issues.

[24] Meyer JP, et al. Leveraging the U.S. criminal justice system to access women for HIV interventions. AIDS Behav. 2017;21(12):3527–48. 10.1007/s10461-017-1778-6 PMC5699977 28534199

[25] Belenko S, Hiller M, Hamilton L (2013). Treating substance use disorders in the criminal justice system. Curr Psychiatry Rep.

[26] Haley DF (2014). Multilevel challenges to engagement in HIV care after prison release: a theory-informed qualitative study comparing prisoners’ perspectives before and after community reentry. BMC Public Health.

[27] Painter TM (2014). Community-based program to prevent HIV/STD infection among heterosexual black women. MMWR Suppl.

[28] Alemagno SA (2009). Brief motivational intervention to reduce HIV risk and to increase HIV testing among offenders under community supervision. J Correct Health Care.

[29] Prendergast M (2011). A multi-site, randomized study of strengths-based case management with substance-abusing parolees. J Exp Criminol.

[30] Brown R (2013). Community-based treatment for opioid dependent offenders: a pilot study. Am J Addict.

[31] Gordon MS (2013). Rapid HIV testing for individuals on probation/parole: outcomes of an intervention trial. AIDS Behav.

[32] Nydegger LA (2013). Effects of a one-hour intervention on condom implementation intentions among drug users in Southern California. AIDS Care.

[33] Lichtenstein B, Barber BW, West Alabama AIDS Outreach Partnership Group (2016). A partnership approach to providing on-site HIV services for probationers and parolees: a pilot study from Alabama, USA. J Int AIDS Soc.

[34] DiClemente RJ (2004). Efficacy of an HIV prevention intervention for African American adolescent girls: a randomized controlled trial. JAMA.

[35] Crepaz N (2009). The efficacy of HIV/STI behavioral interventions for African American females in the United States: a meta-analysis. Am J Public Health.

[36] El-Bassel N (2010). National Institute of Mental Health Multisite Eban HIV/STD Prevention Intervention for African American HIV serodiscordant couples: a cluster randomized trial. Arch Intern Med.

[37] Adimora AA, Schoenbach VJ. Contextual factors and the black-white disparity in heterosexual HIV transmission. Epidemiology. 2002;13(6):707–12. 10.1097/00001648-200211000-00016 12410013

[38] El-Bassel N (1996). Correlates of crack abuse among drug-using incarcerated women: psychological trauma, social support, and coping behavior. Am J Drug Alcohol Abuse.

[39] Epperson MW (2009). Fear, trust, and negotiating safety: HIV risks for black female defendants. Affilia.

[40] Weir BW (2009). Reducing HIV and partner violence risk among women with criminal justice system involvement: a randomized controlled trial of two Motivational Interviewing-based interventions. AIDS Behav.

[41] Kalichman SC (1998). Sexual coercion, domestic violence, and negotiating condom use among low-income African American women. J Womens Health.

[42] El-Bassel N (2001). Correlates of partner violence among female street-based sex workers: substance abuse, history of childhood abuse, and HIV risks. AIDS Patient Care STDS.

[43] Gielen AC (2007). HIV/AIDS and intimate partner violence: intersecting women's health issues in the United States. Traum Viol Abuse.

[44] Mittal M, Senn TE, Carey MP (2012). Intimate partner violence and condom use among women: does the Information–Motivation–Behavioral Skills Model explain sexual risk behavior?. AIDS Behav.

[45] Gilbert L (2015). Targeting the SAVA (substance abuse, violence and AIDS) syndemic among women and girls: a global review of epidemiology and integrated interventions. J Acquir Immune Defic Syndr.

[46] Curran GM (2012). Effectiveness-implementation hybrid designs: combining elements of clinical effectiveness and implementation research to enhance public health impact. Med Care.

[47] Metsch LR (2012). Implementing rapid HIV testing with or without risk-reduction counseling in drug treatment centers: results of a randomized trial. Am J Public Health.

[48] Metsch LR (2013). Effect of risk-reduction counseling with rapid HIV testing on risk of acquiring sexually transmitted infections: the AWARE randomized clinical trial. JAMA.

[49] Bandura A (1977). Self-efficacy: toward a unifying theory of behavioral change. Psychol Rev.

[50] Bandura A (1991). Social cognitive theory of self-regulation. Organ Behav Hum Decis Process.

[51] Peled E (2000). Choice and empowerment for battered women who stay: toward a constructivist model. Soc Work.

[52] Damschroder LJ (2009). Fostering implementation of health services research findings into practice: a consolidated framework for advancing implementation science. Implement Sci.

[53] El-Bassel N (1995). Preventing HIV/AIDS in drug-abusing incarcerated women through skills building and social support enhancement: preliminary outcomes. Soc Work Res.

[54] Schilling RF (1991). Correlates of drug use, sexual behavior, and attitudes toward safer sex among African-American and Hispanic women in methadone maintenance. J Drug Issues.

[55] Bandura A. Perceived Self-Efficacy in Cognitive Development and Functioning, Educational Psychologist. 1993;28(2):117–48. 10.1207/s15326985ep2802_3.

[56] Daveson B (2016). Translating care into outcomes: a grounded theory study using the Consolidated Framework for Implementation Research (CFIR) [abstract PO51]. Palliat Med.

[57] Kirk MA (2016). A systematic review of the use of the Consolidated Framework for Implementation Research. Implement Sci.

[58] Liang S (2016). Integrating evidence-based practices for increasing cancer screenings in safety net health systems: a multiple case study using the Consolidated Framework for Implementation Research. Implement Sci.

[59] Northridge ME (2016). Third places for health promotion with older adults: using the Consolidated Framework for Implementation Research to enhance program implementation and evaluation. J Urban Health.

[60] Birken SA (2017). Combined use of the Consolidated Framework for Implementation Research (CFIR) and the Theoretical Domains Framework (TDF): a systematic review. Implement Sci.

[61] Klafke N, et al. How the Consolidated Framework for Implementation Research can strengthen findings and improve translation of research into practice: a case study. Oncol Nurs Forum. 2017;44(5):E223–31. 10.1188/17.ONF.E223-E231 28820519

[62] VanDevanter N (2017). Application of the Consolidated Framework for Implementation Research to assess factors that may influence implementation of tobacco use treatment guidelines in the Viet Nam public health care delivery system. Implement Sci.

[63] Lewin K (1947). Frontiers in group dynamics: II. Channels of group life; social planning and action research. Hum Relat.

[64] Khan MR (2012). The promise of multimedia technology for STI/HIV prevention: frameworks for understanding improved facilitator delivery and participant learning. AIDS Behav.

[65] Rollnick S, Miller WR (1995). What is Motivational Interviewing?. Behav Cogn Psychother.

[66] Dowding D (2013). Best Practices for Mixed Methods Research in the Health Sciences [book review]. Qual Soc Work.

[67] Russell LB. The science of making better decisions about health: cost-effectiveness and cost-benefit analysis. Presented at the Population Health Workshop sponsored by the Office of Behavioral and Social Science Research, National Institutes of Health, March 26–27, 2014. http://www.sas.rutgers.edu/virtual/snde/wp/2014-06.pdf. Accessed 30 Aug 2017.

[68] Musgrove P, Fox-Rushby J. Cost-effectiveness analysis for priority setting. In Jamison DT, Breman JG, Measham AR, Alleyne G, Claeson M, Evans DB, Jha P, Mills A, Musgrove P, editors. Disease control priorities in developing countries. 2nd ed. Washington, DC: International Bank for Reconstruction and Development/The World Bank Group and New York: Oxford University Press; 2006. 21250354

[69] Pinkerton SD (1998). Toward a standard sexual behavior data set for HIV prevention evaluation. Am J Health Behav.

[70] Cohen DA, Wu SY, Farley TA (2004). Comparing the cost-effectiveness of HIV prevention interventions. J Acquir Immune Defic Syndr.

[71] Goodman LA, Corcoran C, Turner K, Yuan N, & Green BL. Assessing traumatic event exposure: General issues and preliminary findings for the stressful life events screening questionnaire. J Trauma Stress. 1998;11(3):521–542. 10.1023/A:1024456713321. 10.1023/A:1024456713321 9690191

[72] Weathers FW, Litz BT, Herman DS, Huska JA, Keane TM. The PTSD Checklist (PCL): Reliability, validity and diagnostic utility. Paper presented at the Annual Meeting of the International Society for Traumatic Stress Studies; San Antonio. 1993;1993.

[73] Kraanen FL, Vedel E, Scholing A, & Emmelkamp PM. The comparative effectiveness of Integrated treatment for Substance abuse and Partner violence (IStoP) and substance abuse treatment alone: a randomized controlled trial. BMC Psychiatry. 2013;13:189. 10.1186/1471-244X-13-189. 10.1186/1471-244X-13-189 PMC3716952 24059784

[74] Strauss MA, Hamby SL, Boney-McCoy S & Sugarman MA. The Revised Conflict Tactics Scales (CTS2). Development and Preliminary Psychometric Data. J Fam Issues. 1996;17(3):283–316. 10.1177/019251396017003001

